# Advancing artificial intelligence ethics in health and genomics: lessons from a public survey in South Korea

**DOI:** 10.3389/fgene.2025.1563544

**Published:** 2025-07-09

**Authors:** Jungim Lee, Wonhoo Yoo, Hannah Kim

**Affiliations:** 1 Graduate School of Public Health, Yonsei University, Seoul, Republic of Korea; 2 Asian Institute for Bioethics and Health Law, Yonsei University, Seoul, Republic of Korea; 3 Division of Medical and Health Law, College of Medicine, Yonsei University, Seoul, Republic of Korea

**Keywords:** public perception, artificial intelligence in healthcare, ethical principles, research ethics guidelines, healthcare artificial intelligence ethical principles education

## Abstract

Advances in healthcare and genetics are becoming increasingly integrated with artificial intelligence (AI), offering transformative potential alongside complex ethical challenges. This study aimed to assess public awareness and perceptions of AI ethics in healthcare (AI-H) in South Korea, with the ultimate goal of informing the development of research ethics guidelines. A nationwide online survey was conducted from January 10 to 20, 2023, targeting the general public, and 1,002 respondents were recruited through stratified random sampling. The questionnaire explored expectations of AI-H, perceived risks, willingness to share different types of personal data, and the perceived importance of various ethical principles and education targets. A large majority of respondents (84.5%) expressed optimism about the positive impacts of AI-H over the next five years, while only 3.1% anticipated negative consequences. Key concerns included the disclosure of personal information (54.0%), potential AI errors causing harm (52.0%), and ambiguous legal responsibilities (42.2%). Willingness to share data was highest for electronic medical records (72.8%), lifestyle data (72.3%), and biometric data (71.3%), while genetic data was least preferred (64.1%). Ethical principles considered most important were privacy protection (83.9%), safety and security (83.7%), legal duties (83.4%), and responsiveness (83.3%). Developers (70.7%), medical institution managers (68.2%), and researchers (65.6%) were identified as top priorities for ethics education, whereas the general public (31.0%) and students (18.7%) ranked lower. This study represents the first nationwide assessment of public ethical awareness of AI-H in South Korea. While there is strong support for AI-H, significant concerns remain, particularly regarding data privacy and legal accountability. The findings highlight the need for expanded ethics education, especially among younger populations, and for balanced attention to ethical principles beyond privacy, such as inclusiveness and accessibility. These insights provide valuable guidance for developing socially responsible AI policies and practices in healthcare.

## 1 Introduction

The rapid advancement of AI technology is impacting various fields, notably healthcare, and has drawn global focus to its ethical, social implications. In response, international organizations such as the World Health Organization (WHO) and the United Nations Educational, Scientific and Cultural Organization (UNESCO) have established ethical guidelines for AI (World Health Organization, 2024). In 2024, WHO released guidance on the ethics and governance of AI in healthcare, focusing on large multimodal models (LMMs) and providing over 40 recommendations for government, technology companies, and healthcare providers to ensure the appropriate use of AI to promote and protect public health. Similarly, 2021 *Recommendation on the Ethics of Artificial Intelligence* of UNESCO led to set global ethical standard emphasizing the protection of human rights and dignity, transparency, accountability, and the rule of law in AI applications. These efforts reflect the growing recognition of the impacts of AI on public health and the need for ethical governance.

Public perception of AI plays a pivotal role in its development and regulation, influencing research funding, policymaking and commercialization (Ada Lovelace Institute, 2023). Understanding public sentiment on AI ethics is essential for guiding developers and policymakers in generating trustworthy and ethically sound AI systems. This is particularly relevant when navigating potential conflicts between ethical principles at different stages of AI application development. Recognizing the significance of public awareness, previous survey studies have been conducted globally to assess public understanding of AI advancements and their ethical implications. Studies from Germany, the Netherlands, the United States, and Japan have explored public perceptions of AI applications in medicine, revealing a mix of optimism and concerns (Fritsch et al., 2022; Yakar et al., 2022; Ikkatai et al., 2022; Kelly et al., 2019).

In South Korea, research on AI in healthcare (AI-H) has been expanding, but there is a notable gap in studies focusing on the general public’s perceptions. While many studies have explored AI-related issues among specific groups, such as healthcare professionals, few have centered on the broader views of the public. For instance, studies by Shin et al. (2017), Park and Shin (2017), Kim (2019), and Kim and Bae (2024) have examined attitudes toward AI among healthcare workers or students, revealing optimism alongside concerns about privacy violations, technological errors, and bioethics. Additionally, recent studies have investigated the role of ethics education in improving AI awareness, underscoring the importance of robust ethical frameworks to effectively address emerging concerns (Ko and Leem, 2021; Kwon and Yang, 2024; Kang, 2024; Jeon et al., 2024).

Despite these ongoing efforts, a significant gap remains between the growing importance of ethical considerations in AI-H and the lack of empirical research on how the general public perceives these developments. Existing studies have largely focused on specific professional groups, leaving the perceptions, understanding, and trust of the general public in AI-H applications underexplored. As interest in establishing ethical standards for AI research and development in healthcare grows, it becomes increasingly important to assess how these standards are recognized and accepted by the broader public. This study aims to address this gap by focusing specifically on the general public’s perceptions of AI-H in South Korea.

Corporate and industrial trends reveal challenges in AI awareness and ethics, with corporate awareness remaining notably limited despite growing interest in AI technologies. The 2020 Corporate Awareness Survey by the Korea Development Institute (KDI) revealed that only 3.6% of companies expressed an intention to adopt AI technologies and solutions (Korea Development Institute, 2020). However, there is significant optimism about AI’s potential impact in the medical and health sectors, highlighting the need for further development and research support. In 2021, a survey conducted by the Presidential Fourth Industrial Revolution Committee found that 99.3% of respondents were aware of AI, with 59.8% expressing high interest in AI technology, particularly among older age groups, indicating a high level of expectation for AI in healthcare from the elderly (Ministry of Interior and Safety, 2021). A more recent domestic consumer survey on generative AI (Mezzomedia, 2023) revealed mixed trust levels, with 56.0% rating trust in generative AI chatbots as *average* and 29.0% expressing trust, while concerns about hallucination, data security, privacy, and unauthorized use of copyrighted materials reflected the complex public sentiment toward AI.

Given these gaps, this nationwide study aims to evaluate public awareness of healthcare AI ethics in South Korea. By analyzing public perceptions, examining the influence of sociodemographic factors, and identifying key ethical concerns, based on the AI development and applications, the research seeks to contribute to the development of ethical guidelines and policy recommendations.

## 2 Materials and methods

### 2.1 Survey methods

An online survey was conducted to assess public awareness and understanding of AI-H ethics and ethical principles in South Korea. The survey was managed by Nielsen Korea, a professional survey firm, and ran for 11 days, from January 10 to 20, 2023. It targeted adults aged 19 and older across 17 regions of the country, using a stratified proportional distribution method based on region, sex, and age. A survey link was emailed to a random sample of 2,824 people from web-based master panel of Nielsen. Of these, 2,231 accessed the survey (participation rate: 79.0%), and 1,002 completed it (completion rate: 44.9%). Respondents received 100 points (equivalent to 100 KRW or 0.08 USD) upon completion. To ensure data reliability, Nielsen Korea applied IP and ID duplication controls through its master panel system thereby guaranteeing that each individual responded only once.

### 2.2 Questionnaire development

The questionnaire was developed with references to several key sources, including the United Kingdom AI awareness survey (Gov.UK, 2019), the AI use awareness survey in South Korea (Presidential Committee on the Fourth Industrial Revolution, 2021), the public awareness survey on digital healthcare (Korea Development Institute, 2021), the WHO artificial intelligence ethics guidelines (World Health Organization, 2021b), and the UNESCO Recommendations on the Ethics of Artificial Intelligence (United Nations Educational Scientific and Cultural Organization, 2021). The researchers analyzed and synthesized findings from these sources to design the questionnaire structure and content. After drafting the initial version, it was reviewed by six co-investigators. A pilot survey was then conducted, and feedback was used to finalize the questionnaire. Although formal control questions were not included, the survey platform applied automated quality control measures, such as response time monitoring and consistency filters, to ensure data reliability.

The questionnaire comprised 10 questions on AI-H awareness, 11 on AI-H ethical principles, and demographic questions (see Data Sheet 1). It incorporated values from the WHO’s six key groups and UNESCO’s four values and ten principles. Question types included closed-ended, free-response, and Likert scales (4-, 5-, and 11-point). Socio-demographic data were analyzed to explore their influence on public awareness of AI ethics in healthcare.

### 2.3 Ethics approval

The survey was approved by the Institutional Review Board (IRB) of Yonsei University (Approval No. Y-2022-1460). In compliance with the Bioethics and Safety Act, the IRB waived the requirement for written consent. Instead, respondents were provided with detailed survey information before starting the online survey, and their voluntary participation was considered as implied consent.

### 2.4 Statistical analysis

Statistical analyses were conducted using t-tests and ANOVA in IBM SPSS 20 software, with visualizations created in Python 3.13.0. Demographic variables, including age, sex, and region of residence, and socio-demographic factors, encompassing education, household income, use of wearable devices and health apps, and engagement with social networking site (SNS), recent medical visit (see Table 1). The survey program enforced mandatory responses, resulting in a complete dataset with no missing values. Scale items were scored from 20 to 100, excluding a baseline score of 0. For instance, on a 5-point scale, response ranged from 20 (very positive) to 100 (very negative).

**TABLE 1:** Socio-demographic characteristics.

Categories	Variables	*n* (%)	Categories	Variables	*n* (%)
Sex	M	503 (50.3)	Education	≤Middle school	14 (1.4)
	F	499 (49.9)		High school	209 (20.9)
Age	19–29	167 (16.7)		College	673 (67.2)
	30–39	160 (16.0)		≥Graduate school	106 (10.6)
	40–49	195 (19.5)	Medical Visits (Yr)	≤1 time	98 (9.8)
	50–59	205 (20.5)		2–5 times	480 (47.9)
	≥60	275 (27.4)		6–10 times	269 (26.8)
Residence	Metro	512 (51.0)		≥11 times	115 (15.5)
	Non-metro	490 (49.0)	Wearable Use	Yes	603 (60.2)
Household Income (USD)	<1,600	77 (7.7)		No	339 (39.8)
	1,600–3,200	247 (24.7)	Health App Use	Yes	755 (75.3)
	3,200–4,800	320 (31.9)		No	247 (24.7)
	≥4,800	330 (32.9)	SNS Use	Yes	933 (93.3)
	Not sure/Rejected	28 (2.8)		No	69 (6.9)

## 3 Results

### 3.1 Socio-demographic characteristics

The 1,002 respondents selected via e-mail had a maximum sampling error of ±3.10 percentage points at a 95% confidence level (see Table 1). The sex distribution was nearly equal, with 503 men (50.2%) and 499 women (49.8%). Age group were evenly represented as follows: 167 respondents (16.7%) were in their 19–20s, 160 (16.0%) in their 30s, 195 (19.5%) in their 40s, 205 (20.5%) in their 50s, and 275 (27.4%) were in their 60s or older. By region, 512 respondents (51.0%) were from metropolitan areas, and 490 (49.0%) from non-metropolitan areas. Educational attainment was high, with 77.8% holding a college degree or above. Monthly household income was broadly distributed, though skewed toward higher brackets. This reflects a common limitation of web-based panel surveys, which tend to overrepresent digitally literate and socioeconomically advantaged individuals. (Bethlehem, 2010; Couper, 2000).

Regarding ownership of portable electronic devices, 365 respondents (36.4%) own only smartphones, 271 (27.1%) own smartphones, smart watches, and tablets, 220 (22.0%) had smartphones and tablets, 122 (12.2%) had smartphones and smart watches, and 23 (2.3%) owned other combinations. In terms of wearable device use, 603 respondents (60.2%) reported having used them, while 399 (39.8%) had not. For health-related app usage, 755 respondents (75.3%) had experience using such apps, while 247 (24.7%) had none. SNS usage was reported by 933 respondents (93.3%), with 69 (6.9%) indicating no usage. The frequency of SNS use varied: 98 respondents (9.8%) used SNS once or less annually, 480 (47.9%) used it 2–5 times, 269 (26.8%) used it 6–10 times, and 155 (15.5%) used more than 11 times. Regarding visits to medical institutions per year, 98 (9.8%) visited once or less, 480 (47.9%) visited 2 to 5 times, 269 (26.8%) visited 6 to 10 times, and 155 (15.5%) visited 11 or more times.

### 3.2 Awareness of AI-H according to respondent characteristics, awareness of AI-H ethical principles, and awareness of the necessity for AI-H ethical principles

Awareness of AI-H, awareness of AI-H principles, and necessity for AI-H principles (excluding 34 ‘unknowns’ responses) were measured using a 4-point Likert scale ranging from ‘very positive’ to ‘very negative’. T-tests were performed for variables such as sex, residence, and experience with wearable device, health-related apps, and SNS. ANOVA was applied for age, income, education, and medical visits. A *p*-value of less than 0.05 was considered statistically significant.

Average differences across these variables were examined to assess their influence on AI-H awareness, ethical principles recognition, and its perceived necessity, as summarized in Table 2.

**TABLE 2:** Awareness of AI-H and ethical principles by socio-demographic factors.

		Awareness of AI-H			Awareness of AI-H ethical principles			Need of AI-H ethical principles		
		*n*(M)	SD	*p*-value	*n*(M)	SD	*p*-value	*n*(M)	SD	*p*-value
Sex	M	503 (2.32)	0.79	0.071	503 (2.37)	0.78	0.427	489 (1.61)	0.68	0.956
	F	499 (2.41)	0.80		499 (2.33)	0.78		479 (1.62)	0.66	
Age	19–29	167 (2.32)	0.90	0.361	160 (1.33)	0.47	0.389	160 (1.81)	0.73	***<0.001***
	30–39	160 (2.33)	0.87		156 (1.39)	0.49		156 (1.70)	0.64	
	40–49	195 (2.39)	0.832		187 (1.41)	0.49		187 (1.59)	0.70	
	50–59	205 (2.45)	0.710		198 (1.43)	0.50		198 (1.53)	0.62	
	≥60	275 (2.32)	0.710		267 (1.40)	0.49		267 (1.53)	0.65	
Residence	Metro	512 (2.33)	0.78	0.176	512 (2.35)	0.78	0.979	499 (1.59)	0.66	0.263
	Non-metro	490 (2.40)	0.81		490 (2.35)	0.78		469 (1.64)	0.68	
Household Income (USD)	<1,600	77 (2.69)	0.88	***<0.001***	77 (2.64)	0.79	***0.002***	77 (1.56)	0.73	***0.001***
	1,600–3,200	247 (2.41)	0.81		247 (2.38)	0.74		247 (1.72)	0.66	
	3,200–4,800	320 (2.27)	0.73		320 (2.28)	0.74		320 (1.64)	0.67	
	≥4,800	330 (2.30)	0.79		330 (2.28)	0.83		330 (1.50)	0.64	
Education	≤High school	223 (2.59)	0.88	***<0.001***	223 (2.45)	0.75	0.072	207 (1.73)	0.69	***0.012***
	College	673 (2.32)	0.75		673 (2.32)	0.78		656 (1.59)	0.65	
	≥Graduate School	106 (2.17)	0.76		106 (2.34)	0.84		105 (1.54)	0.73	
Medical Visits (Yr)	≤1 time	98 (2.63)	0.75	***<0.001***	98 (2.49)	0.65	***0.014***	88 (1.68)	0.64	0.354
	2–5 times	480 (2.42)	0.77		480 (2.39)	0.77		466 (1.62)	0.67	
	6–10 times	269 (2.24)	0.79		269 (2.31)	0.80		262 (1.63)	0.69	
	≥11 times	155 (2.24)	0.85		155 (2.20)	0.83		152 (1.53)	0.69	
Wearable Use	Yes	603 (2.17)	0.76	***<0.001***	603 (2.16)	0.78	***<0.001***	594 (1.60)	0.70	0.416
	No	399 (2.65)	0.76		399 (2.63)	0.69		374 (1.64)	0.63	
Health App Use	Yes	755 (2.19)	0.74	***<0.001***	755 (2.20)	0.77	***<0.001***	747 (1.58)	0.66	***<0.001***
	No	247 (2.88)	0.74		247 (2.80)	0.63		221 (1.75)	0.71	
SNS Use	Yes	933 (2.33)	0.78	***<0.001***	933 (2.32)	0.78	***<0.001***	912 (1.61)	0.67	0.121
	No	69 (2.83)	0.84		69 (2.75)	0.65		56 (1.75)	0.75	

Bolded p-values indicate statistically significant differences (p < 0.05).

There was no statistically significant difference in the average level of AI-H awareness, awareness of ethical principles, and the perceived necessity of these principles based on sex and region of residence.

However, a significant difference was observed in the perceived necessity for AI-H ethical principles across age group (*p* < 0.05). The lowest average ratings—indicating the highest perceived necessity—were observed among respondents in their 50s and those aged 60 or older. These values showed a clear upward trend with decreasing age, indicating that younger respondents were generally less likely to recognize the importance of ethical principles in AI-H.

Awareness of AI-H was highest among those earning 3,200–4,800 USD. For awareness of AI-H ethical principles, the highest levels were observed both in the 3,200–4,800 USD group and among those earning more than 4,800 USD. In terms of perceived necessity, the highest level was reported by respondents with a monthly income above 4,800 USD.

Educational background was significant associated with both AI-H awareness and the perceived necessity of ethical principles (*p* < 0.05). Awareness of AI-H was highest among those enrolled in graduate school or higher, followed by college graduates and then high school graduates. Likewise, awareness of the need for AI-H ethical principles was highest among those enrolled in graduate school or higher, followed by college graduates and high school graduates. This pattern showed that higher educational attainment is positively correlated with better awareness of AI-H and the perceived importance of AI-H ethical principles.

The frequency of visits to medical institutions within the past year also influenced awareness level (*p* < 0.05). The groups who visited medical institutions 11 or more times and those who visited 6–10 times were the most aware of AI-H, followed by those with 2–5 visits, and lastly, those with 1 or fewer visits. These findings indicate that more frequent interaction with healthcare services is associated with higher awareness of AI-H and the importance of its ethics.

Finally, the group with no experience using wearable devices, health-related apps, or SNS was less aware of AI-H and its ethical principles compared to the group with experience (*p* < 0.05). Regarding the need for AI-H ethical principles, the group with experience using healthcare apps was more positive about the need for AI-H ethical principles compared to the group with no experience using them (*p* < 0.05).

### 3.3 Expected benefits and concerns of AI-H in the next 5 years

Most respondents expected AI-H to have a positive impact over the next 5 years, with 84.5% expressing this view, while only 3.1% anticipated negative effects. The top three expected benefits were: identifying the causes of diseases such as cancer and rare conditions (52.4%), developing treatments for these diseases (43.0%), and improving access to emergency personal electronic medical records (42.7%).

In contrast, among the 3.1% who expressed concerns, the most frequently cited issues were: disclosure of personal and sensitive health information (54.0%), harm from diagnostic errors (52.0%), and ambiguity in legal responsibilities for AI-related damage (42.2%).

**FIGURE 1:**
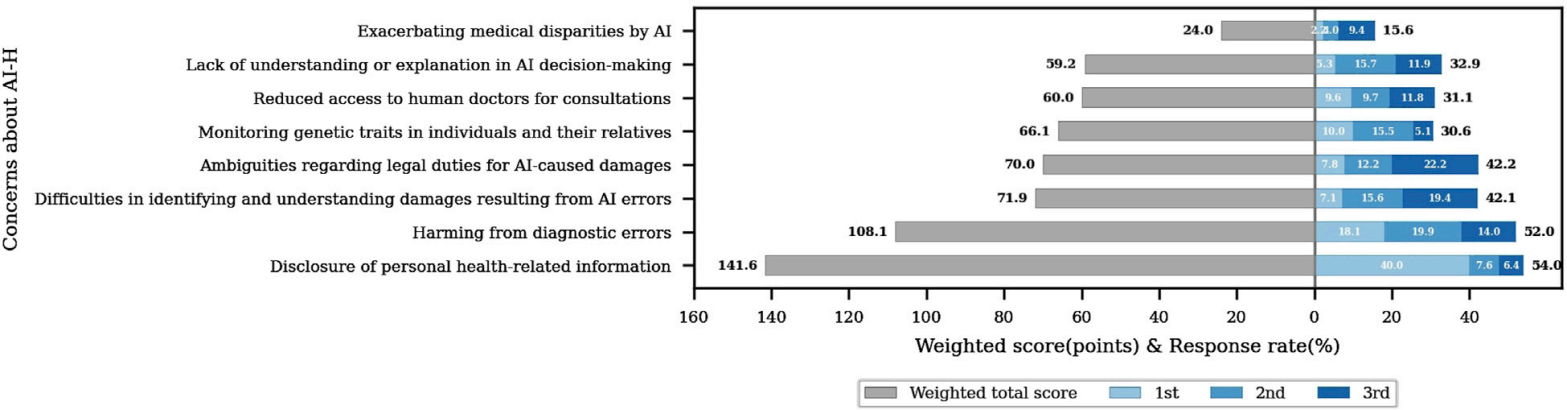
Types of concerns about AI-H over the next 5 years based on weighted ranking.

These concerns are summarized and presented in Figure 1, which displays their relative importance based on weighted scoring. Respondents were asked to select their top three concerns related to the future of AI in healthcare. Weighted scores were calculated by assigning 3 points to first-ranked concerns, 2 points to second-ranked, and 1 point to third-ranked concerns. The grey bars represent the total weighted scores, while the blue shades indicate the proportion of respondents who selected each concern as their first, second, or third priority. Weighted score (points) and Response rate (%) are shown on the horizontal axis.

Based on this method, Figure 1 shows that disclosure of personal health information received the highest score (141.6), followed by harm from diagnostic errors (108.1), and difficulty in recognizing AI-related harm (71.9). Other concerns included ambiguity in legal duties (70.0), genetic monitoring (66.1), reduced access to human doctors (60.0), lack of explanation in AI decisions (59.2), and widening medical disparities (24.0).

These findings suggest that data privacy is perceived by the Korean public as the most urgent ethical concern regarding AI in healthcare.

### 3.4 Preferences for genetic and health data sharing

When asked about their willingness to use AI-H technology in the future, most respondents showed a positive response (79.6%), with 20.1% strongly agreement and 59.5% agreement. Participants were then asked about their willingness to provide several types of personal data with third parties for the future use of AI-H. Over half of the respondents expressed willingness to share their data.

**FIGURE 2:**
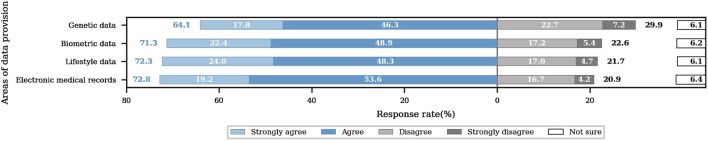
Preferences to share individual data sharing in future AI-H development.

As shown in Figure 2, response rates are presented across five categories—Strongly agree, Agree, Disagree, Strongly disagree, and Not sure—across four data types: electronic medical records, lifestyle data, biometric data, and genetic data. Support for data sharing was highest for electronic medical records (72.8%), followed closely by lifestyle data from smartphones or wearables (72.3%) and biometric data (71.3%). In contrast, willingness to share genetic data was lower (64.1%), likely reflecting greater privacy concerns.

Among these, genetic data was the type participants were most reluctant to provide. Among those hesitant to provide genetic data, the most cited concerns were: the disclosure of personal health-related information (39.3%), the potential harm caused by AI diagnostic errors (18.5%), and monitoring the genetic traits of individuals and their relatives (9.9%). Notably, both the concern about health data disclosure and the monitoring of genetic traits reflects a common underlying issue, the protection of personal privacy. Both concerns highlight the sensitivity surrounding genetic data and the importance of safeguarding personal information in the context of AI-H applications.

### 3.5 Necessity for ethical education among AI stakeholders

When asked whether they had heard of AI-H ethical principles, 60.5% of respondents indicated familiarity, while 39.4% were not aware of them. Additionally, 89.6% of respondents recognized the necessity of ethical principles in the development and use of AI-H, whereas only 7.0% considered them unnecessary.

**FIGURE 3:**
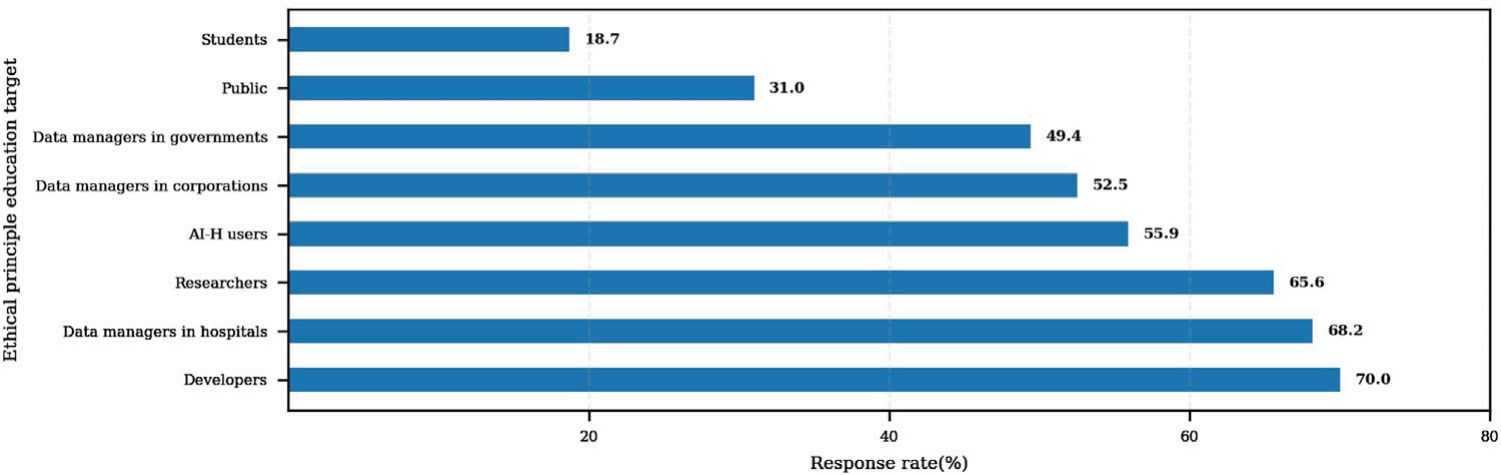
Priority groups for ethical principles education for AI-H.

To address these needs, respondents identified key stakeholder groups for ethics education and multiple responses were allowed. Developers were selected by 70.0% of participants, followed by healthcare administrators (68.0%), researchers (66.0%), and users (56.0%). Figure 3 displays the percentage of respondents who selected each group, highlighting developers, administrators, and researchers as the top priorities for ethics education.

In this study, 17 ethical principles were identified based on the World Health Organization, (2021a) and United Nations EducationalScientific and Cultural Organization, (2021) ethical guidelines as well as a comprehensive literature review. Survey participants rated the importance of each principle on a 10-point scale, with 1 being very unimportant and 10 being very important. The ratings were subsequently converted to a 100-point scale for analysis.

**FIGURE 4:**
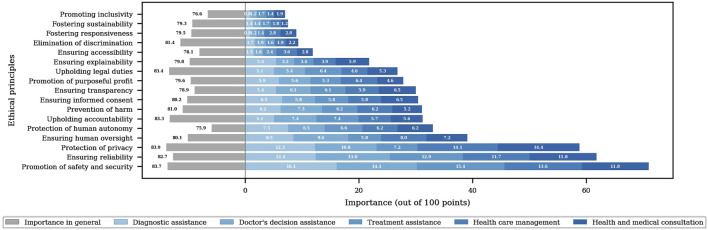
Importance of ethical principles in AI-H across different cases.

As shown in Figure 4, grey bars indicate the overall importance scores for each ethical principle, while blue segments represent how importance varied across specific AI-H application contexts: diagnostic assistance, doctor’s decision assistance, treatment assistance, healthcare management, and health and medical consultation.

The 17 ethical principles included: promotion of safety and security, ensuring reliability, protection of privacy, ensuring human oversight, protection of human autonomy, upholding accountability, prevention of harm, ensuring transparency, ensuring informed consent, promotion of purposeful profit, upholding legal duties, ensuring explainability, ensuring accessibility, elimination of discrimination, fostering responsiveness, fostering sustainability, and promoting inclusivity.

Among these, protection of privacy was rated highest at 83.9 points, followed by promotion of safety and security (83.7), upholding legal duties (83.4), and upholding accountability (83.3).

The top three ethical principles prioritized for each AI use case were as follows: for diagnosis assistance AI—promotion of safety and security (16.1%), ensuring reliability (12.4%), and protection of privacy (12.3%); for doctor’s decision assistance AI—promotion of safety and security (14.1%), ensuring reliability (13.0%), and protection of privacy (10.8%); for treatment assistance AI—promotion of safety and security (15.4%), protection of privacy (12.9%), and ensuring human oversight (9.6%); for healthcare management AI—protection of privacy (14.1%), promotion of safety and security (13.6%), and ensuring reliability (12.7%); for healthcare consultation AI—protection of privacy (14.4%), promotion of safety and security (11.8%), and ensuring reliability (11.7%).

Overall, ethical principles for each use case of AI-H were considered most important in the order of promoting safety and security, ensuring reliability, and protecting privacy. In contrast, principles such as promoting inclusivity, ensuring accessibility, eliminating discrimination, fostering responsiveness, and ensuring sustainability were rated as lower priorities.

## 4 Discussion

### 4.1 Recognizing public awareness to strengthen ethical standards for AI-H

The medical field utilizing AI continues to invest in research aimed at enhancing medical professionals’ insights, improving treatment strategies, and delivering higher-quality medical services to patients. To support this progress, a survey was conducted to evaluate South Korean awareness of AI-H utilization and ethical principles. The key discussion points are as follows.

Establishing and updating ethical guidelines in response to public awareness is critical to ensuring the safe use of AI-H. It is essential to develop guidelines that integrate technical standards with ethical considerations, addressing public concerns and fostering trust. So far, awareness surveys on AI in the medical field have been conducted among patients (Young et al., 2021; Khullar et al., 2022), medical students (Chen et al., 2022; Teng et al., 2022; Mehta et al., 2021), and medical expert groups in the United Kingdom (Castagno and Khalifa, 2020), Germany (Maassen et al., 2021), and France (Laï et al., 2020), with findings generally indicating positive reception.

In this study, public perception was positive at 84.5%, representing a slight increase from 81.9% in the 2021 KDI survey and a higher proportion compared to 53.1% in the German survey by Fritsch et al. (2022). Conversely, in the United States, more than half of the population recently expressed negative opinions (Pew Research Center, 2023) and reported concerns about discomfort, anxiety, and uncertainty in a study by Rojahn et al. (2023).

This study found that Koreans expect AI to be utilized in the medical field, including identifying the causes of cancer and rare diseases, developing treatments, enhancing the usability of electronic medical record (EMR) web systems, and improving the accuracy of medical image interpretation. In a study by Kwon (2022), it was expected that AI would improve the treatment process, predict and prevent personal diseases, and enable precise diagnosis and treatment. Fast and Horvitz (2017) indicated that AI would enable diagnosis, new drug development, and customized medical support. Similarly, Beets et al. (2023) highlighted that AI would significantly impact disease prediction, diagnosis, treatment, and care.

On the other hand, concerns were ranked in the order of personal data disclosure, such as health information, diagnostic errors, malfunctions, and legal duties. Kwon (2022) noted difficulties in emotional interaction and the risk of accidents due to malfunctions. A study by Esmaeilzadeh (2020) also highlighted issues related to the protection of privacy, technical challenges in implementing AI, moral dilemmas, and discrimination. Additionally, a study by Wittal et al. (2022), conducted by a group of German medical experts, raised concerns about the exposure of personal information.

Overall, while this study confirms the widespread optimism about AI’s potential to enhance medical diagnosis, treatment, and personalized care, it also highlights significant concerns related to privacy, technical reliability, and ethical dilemmas. These findings are consistent with previous research, suggesting that while the benefits of AI in healthcare are widely recognized, addressing these concerns is crucial for developing ethical guidelines and fostering public trust in AI-H.

### 4.2 Emphasizing privacy and safety as core ethical priorities in AI-H development

In this study, 79.5% of Koreans indicated a willingness to use AI technology in the medical field and to provide their information for future healthcare AI technology (72.8% for electronic medical records, 72.3% for lifestyle data, and 71.3% for biometric data). The demand for AI utilization in healthcare is increasing, and the rapid transformation of the market is influencing its development direction. A comprehensive guidelines model is essential, encompassing personal information protection and adhering to technical and ethical standards to ensure a safe future medical environment.

Reflecting public awareness in policies is equally important. Ethical guidelines and policies must be regularly updated to align with evolving public perceptions and understanding of AI technologies across various applications. The survey results demonstrated that ethical principles related to data management, such as quality assurance and security protocols, were considered highly important, indicating a growing awareness of the potential risks and benefits associated with data handling. This emphasis on data management aligns with the concerns about responsibility, diagnostic errors, and data breaches raised in the 2021 KDI Digital Healthcare National Survey.

The findings of this study highlight that privacy protection emerged as a critical theme, receiving greater emphasis compared to previous surveys. This is evidenced by two key results: first, the primary reason for reluctance to provide genetic data for AI-H technology was the risk of personal information leakage (39.3%); second, among the 17 ethical principles, privacy protection was rated as the most important (83.9 points). These results provide significant policy implications for the development of AI-H, underscoring the need to prioritize privacy and data security. This aligns with recent findings by Özkan et al. (2022) and Thomas et al. (2024), who highlight growing public concerns over the vulnerability of genetic data and the inadequacy of existing safeguards to ensure privacy. Despite existing legal protections for personal and genetic data, it remains essential to evaluate whether public concerns arise from substantive risks or can be addressed through informed dialogue (Personal Information Protection Act; Medical Service Act; Bioethics and Safety Act Art.46).

In clinical scenarios directly impacting life—such as diagnostic support AI, physician decision support AI, and treatment support AI—the top priorities were identified as ensuring safety and security, reliability, and privacy protection. In comparison, for health promotion purposes—such as healthcare management and medical consultation—the priorities shifted to privacy protection, safety and security, and reliability. This suggests that in South Korea, AI-H ethics are prioritized differently based on the context: safety and security are emphasized in critical life-sustaining scenarios, while privacy protection takes precedence in preventive healthcare.

A recent data breach in Korea has intensified privacy concerns, emphasizing the need to establish public trust before using sensitive data in AI research. Without ethical safeguards, such concerns may lead to public opposition (Lee and Lee, 2025; Korea JoongAng Daily, 2025). The quantity and quality of data are crucial for promoting medical innovation (Stanfill and Marc, 2019). In South Korea, ongoing institutional improvements are being made to facilitate the utilization of big data (Ministry of Health and Welfare, 2024; Choi and Kim, 2022). To support these efforts, researchers, companies, and governments must strengthen their awareness of healthcare AI ethics (Wylie, 2020; Atenas et al., 2023). Continuous and proactive policy adjustments are needed to ensure that ethical principles are effectively integrated into AI-H development. At this stage, it is essential to establish and evaluate policies that reflect the public perception of ethical principles in AI-H research and development.

### 4.3 Aligning policy and innovation for ethical AI-H implementation

This study confirmed that awareness of AI-H and ethical principles increased in parallel to the rise in use of wearable devices, healthcare apps, SNS, and frequent visits to medical institutions (*p* < 0.05). South Korea’s AI technology has become popular as a result of significant advancements in cutting-edge technology (OECD, 2023).

In the 2020 Oxford Insight evaluation, South Korea rose from 26th to 7th place among 172 countries compared to the previous year, demonstrating its preparedness for AI adoption. This improvement was mainly attributed to the government’s strategic vision and active policy promotion (100 points), along with strong performance in Governance and Ethics (85.6 points), which had a positive impact (European Union, 2020).

Domestically, notable changes in healthcare policies have also been observed. The EMR certification system (Medical Service Act Art. 23, Section 2, Paragraph 1) was newly implemented following the 2016 revision of the Medical Act. Interest in AI medical applications grew further with the introduction of IBM Watson in some domestic hospitals beginning in 2016. Recently, selective application of national health insurance to AI medical devices, particularly in the field of radiology, has also been attempted. Additionally, the number of direct doctor consultations per person per year is 7.2 in South Korea, compared to the OECD average of 6.8 (Organization for Economic Co-operation and Development, 2021). This indicates that high accessibility to medical care may provide context for the findings of this study.

However, under the South Korean Medical Device Act, healthcare apps are regulated by this Act only if they fulfill the purpose of diagnosing, treating, alleviating, managing, or preventing diseases (applicable only to ‘mobile medical apps’) (Art. 2). In contrast, apps that provide general medical information, support self-health management, assist OCS or EMR systems on mobile devices, facilitate medical examinations through interviews for medical professionals and patients, or offer communication systems such as video support for consultations between medical personnel and patients are not subject to regulation under the Medical Device Act (Ministry of Food and Drug Safety, 2020).

The need for strengthening and expanding ethical guidelines has been emphasized by various groups, including scientists (Lee et al., 2021), legal scholars (Gerke et al., 2020), and experts (Ministry of Science and ICT, 2018; Borenstein and Howard, 2021; Naik et al., 2022). Additionally, the World Health Organization, (2021b) highlighted the importance of adhering to ethical principles when implementing technologies in digital health.

The finding that healthcare app users show a high awareness of the need for ethical principles is particularly significant. Although advancements in technology are driving changes in healthcare policy, there is a growing demand for the broader application of ethical principles among healthcare app users. Therefore, public-private partnerships are needed to develop and expand related regulations and ethical guidelines.

### 4.4 Expanding ethics education for AI-H stakeholders and the public

Significant differences in awareness of AI-H and its ethical principles were observed across age groups and educational backgrounds, with younger individuals and those with lower education levels showing notably lower awareness. The fact that the younger the age group, the less aware they are of the need for ethical principles is a serious concern. Ethical values are not innate but are cultivated through education (Grusec et al., 2014; Park, 2020). Dabbagh et al. (2024) stated that AI ethics education should be mandatory from an early age to establish an ethical foundation; Kim (2020) emphasized that both human and AI ethics should be included in moral education curricula.

Several countries have already established national AI ethics education programs. Finland has developed a national AI ethics curriculum, which has been available to the public since 2020 (University of Helsinki, 2018). In Germany, the federal government has focused on public education by presenting AI ethics guidelines and policy recommendations since 2019 (European Union, 2018). In South Korea, following the announcement of the National AI Strategy in 2021, AI ethics will be incorporated into the school curriculum starting in 2025 (Ministry of Science and ICT, 2019).

According to the survey results, those identified as needing education on ethical principles include a high proportion of developers (70.7%), medical institution managers (68.2%), researchers (65.6%), and users (55.9%). Health app users also showed a strong recognition of the need for AI-H ethical principles (*p* < 0.05), which may be attributed to their closer engagement with healthcare technologies. This finding aligns with the fact that developers, medical managers, and researchers—who also work directly with such technologies—were identified as key targets for ethics education. These overlapping results support the need for targeted educational strategies focused on these key stakeholder groups.

In contrast, the proportions are significantly lower among students (18.7%) and the general public (31.0%). In South Korea, while advanced AI technology is widely adopted, public awareness of the necessity of ethics education remains relatively low. These findings highlight the need for more inclusive and diversified ethics education efforts, especially among groups with lower levels of awareness. AI ethics education requires diverse approaches and curricula to help scholars, scientists, and citizens recognize ethical issues and their significance (Akgun and Greenhow, 2022).

### 4.5 Awareness gaps for equitable and inclusive AI-H adoption

World Health Organization, (2021a) presented AI ethics guidelines with six core principles centered on ethics and human rights. These guidelines emphasize that AI can expand access to medical services for underprivileged groups, improve public health surveillance, and enable healthcare providers to better serve patients. To achieve universal healthcare, the WHO also stressed the importance of eliminating biases based on race, ethnicity, age, and gender, as well as bridging the digital divide.

However, in this study, principles such as ensuring accessibility (11.9%), eliminating discrimination (9.4%), fostering responsiveness (7.9%), fostering sustainability (7.5%), and promoting inclusivity (7.0%) ranked significantly lower compared to promoting safety and security (71.0%), ensuring reliability (62.7%), protecting privacy (58.8%), and ensuring human oversight (39.1%). This indicates a disproportionate awareness of AI ethical principles in South Korea.

Katirai (2023) highlighted that Japan’s AI-H development has overlooked key ethical concerns, particularly responsiveness and sustainability. Similarly, South Korea, as it transitions into a multicultural society with foreign residents comprising 4.9% of the population (Ministry of Justice, 2023), faces challenges in promoting diversity and equity.

To mitigate bias, education has been identified as a key policy tool (McBride, 2015). AI ethics education should adopt a human rights-based approach from early childhood (Raabe and Beelmann, 2011; Wong, 2020). Guidelines that promote risk awareness and action-oriented diversity policies must be reinforced (Cachat-Rosset and Klarsfeld, 2023). Furthermore, infrastructure development is essential to empower AI developers and users (Seppälä et al., 2021). Comprehensive ethics education programs will be critical in ensuring the ethical and sustainable development of AI-H for future generations.

## 5 Limitations

First, the limitations of the study stem from the sample of respondents. Since the survey was conducted using a panel group from a survey company, generalization is limited due to positive bias and lack of diversity in the results, which may not represent the entire population. Respondents were more likely to provide socially desirable answers due to the survey being conducted via email, and their higher socioeconomic status and knowledge levels (77.7% with a college degree or higher, 64.8% with an income over 3,200 USD) may have influenced the results (Korack-Kakabadse and Korack-Kakabadse, 1999). Future research should aim for a more diverse and representative sample to improve generalizability.

Second, while the public perception survey on AI-H shows positive views about AI-H, expert opinions from medical professionals, lawyers, policymakers, and AI developers should be considered for introducing AI-H. These experts have more experience and insights that can complement public opinion in the development of AI-H policies (Matheny et al., 2020).

Third, further research is needed to explore why principles like accessibility, elimination of discrimination, responsiveness, sustainability, and inclusivity received low rankings. Understanding reasons behind the rankings is crucial for promoting the beneficial use of AI-H, guiding policy decisions, and preventing healthcare inequalities.

## 6 Conclusion

Medicine in South Korea is at a critical juncture where safe and effective use of AI-driven system has become essential. As AI continues to be integrated into healthcare, it is imperative to ensure that AI-H system operates within ethical boundaries, safeguarding patient health, welfare and broader societal interests.

This study, the first of its kind in South Korea, assessed public awareness of AI ethics in healthcare (AI-H) and revealed a combination of high expectations and significant concerns. Most respondents anticipated positive impacts from AI-H in the next 5 years (84.5%), while only 3.1% expected negative effects. However, there were prominent concerns about personal information disclosure (54.0%), AI errors leading to harm (52.0%), and unclear legal responsibilities (42.2%). Additionally, although there was strong agreement on the importance of ethical principles for AI-H (89.6%), an imbalanced perception of ethical priorities was observed: privacy protection was rated highest (83.9%), while inclusiveness (76.6%) and accessibility (78.1%) were rated relatively lower. The findings also indicated low ethical awareness among younger populations and a general consensus that developers, institutional managers, and researchers should be the primary recipients of ethics education, rather than the public or students. These results highlight several pressing challenges, including the need for broader ethics education and the importance of fostering a more balanced understanding of various ethical principles. Addressing these issues is crucial to fostering a well-rounded understanding of AI in medicine. To successfully harness AI-H, the study advocates for policy changes that adopt a multifaceted and multidisciplinary approach, as opposed to a one-size-fits-all solution, ensuring that ethical considerations evolve alongside advancements in AI-H.

As part of the development of the *Research Ethics Guidelines for AI Researchers in Healthcare* on behalf of the KNIH, the findings of the study contributed to the release of these guidelines in 2023. Designed to establish ethical standards for AI researchers and developers in healthcare, the guidelines promote a self-regulatory approach aimed at addressing public concerns over the potential risks of AI-H. However, it is also crucial to identify and address any gaps between public perception of the research findings, and stakeholder viewpoints, as well as the guidelines themselves. Continuous dialogue with diverse stakeholders, including healthcare professionals, policymakers, and the public, is vital to keeping the guidelines relevant, effective, and aligned with evolving social, ethical, and technical standards. By incorporating such feedback, these guidelines can serve as a dynamic framework for the review, evaluation, and responsible implementation of AI research and development in healthcare, ensuring that advancements benefit society and protecting individual rights.

## Data Availability

The raw data supporting the conclusions of this article will be made available by the authors, without undue reservation.
