# Correction: Advancing artificial intelligence ethics in health and genomics: lessons from a public survey in South Korea

**DOI:** 10.3389/fgene.2026.1769636

**Published:** 2026-01-13

**Authors:** Jungim Lee, Wonhoo Yoo, Hannah Kim

**Affiliations:** 1 Graduate School of Public Health, Yonsei University, Seoul, Republic of Korea; 2 Asian Institute for Bioethics and Health Law, Yonsei University, Seoul, Republic of Korea; 3 Division of Medical and Health Law, College of Medicine, Yonsei University, Seoul, Republic of Korea

**Keywords:** public perception, artificial intelligence in healthcare, ethical principles, research ethics guidelines, healthcare artificial intelligence ethical principles education

An **Incorrect number** was provided for the 2025 funding from the “Korea Disease Control and Prevention Agency”. The **Correct number** is “2025-ER0701-00”.

The original article has been updated

