# Supplementary material for: Advancing artificial intelligence ethics in health and genomics: lessons from a public survey in South Korea
Source: Front Genet. 2025 Jul 9;16:1563544. doi: 10.3389/fgene.2025.1563544 (PMC12284414; doi:10.3389/fgene.2025.1563544)
Supplement: Supplementary file 1 [file DataSheet1.pdf]

‘Public Survey on Ethical Principles of AI in Healthcare (AI-H)’

Dear Participant,

We are the Yonsei University Medical Law and Ethics Research Institute, which conducts various research and academic activities to establish legal and ethical standards necessary for the Korean and global healthcare sectors, establish the academic identity of healthcare law and ethics, and systematically research and develop them.

To further this aim, we are conducting a survey to understand public perceptions of applying AI technology in healthcare and attitudes toward related ethical principles. The information you provide will serve as foundational data for future research and policy development related to AI-H. We would greatly appreciate it if you could take a few minutes to participate in this survey.

Estimated survey duration: 20 minutes

Thank you for taking the time to participate in our survey.

Survey Details:

- **Survey Period:** December 2022 – January 2023
- **Target Participants:** General citizens from 17 cities and provinces nationwide
- **Research Institution:** Yonsei University Medical Law and Ethics Research Institute
- **Principal Researcher:** Prof. Han Nah Kim
- **Researcher in Charge:** Assistant Professor Soo-min Kim
- **Researcher in Charge Email:** ksumin91@yuhs.ac
- **Field Survey Company:** Nielsen IQ Korea Ltd.
- **Field Survey Manager:** Jin-woong Kim, Team Manager of Nielsen IQ Korea Social Public Survey Division
- **Field Survey Manager Email:** jinwoong.kim@nielseniq.com

  Confidentiality Notice:

STATISTICS ACT

Article 33 (Protection of Secrets) (1) Matters belonging to the confidential information of individuals, corporations, organizations, etc. that have become known in the course of producing statistics shall be protected.

(2) Data belonging to the confidential information of individuals, corporations, organizations, etc. that have been produced for the production of statistics shall not be used for any purpose other than that of producing statistics.

SQ Selecting respondents

SQ1. What is your age range? (As of January 2023)

① 19-29

② 30-39

③ 40-49

④ 50-59

⑤ 60 or older

SQ2. What is your sex?

① Male

② Female

SQ3. Where do you currently live?

① Seoul

② Gyeonggi-do/Incheon

③ Chungcheong-do/Daejeon/Sejong

④ Gyeongsang-do/Busan/Daegu

⑤ Jeolla-do/Gwangju

⑥ Gangwon-do

⑦ Jeju-do

I. Awareness of AI-H

Artificial Intelligence (AI) is a field of computer engineering and likewise system that perform tasks by imitating human intelligence and improving their performance based on the information they collect.

AI is actively being developed in the healthcare field, with several areas already seeing its application. Notable examples include systems that collect and analyze patient information for health management, and programs that interpret medical images to assist in diagnosis.

In other words, **AI-H** refers to the application and utilization of AI technology across various healthcare and medical domains, including medical research, public health, and clinical practice. Recently, the scope of AI-H has rapidly expanded to encompass areas such as collecting and analyzing patient information, patient treatment, hospital management, and assisting in the diagnosis of medical images like X-rays.

Q1. How familiar are you with 'AI-H'?

① I know it well

② I know it to some extent

③ I've heard of it, but I don't know much of it

④ I've never heard of it

Q2. Have you used any AI-H products or services in the past year? (Please select all that apply)

① Health applications on wearable devices or smartphones (e.g., Apple Health, Samsung Health, etc.)

② Healthcare chatbots (e.g., health-related information is provided through automated response systems such as healthcare consultation AI, messengers, etc.)

③ Websites that display your electronic medical records (e.g., National Health Insurance Corporation's MyData, etc.)

④ Other (please specify):

⑤ Never used any

Q3. Do you agree that AI-H will have positive impact our country's healthcare sector over the next five years?

① Strongly agree

② Agree

③ Slightly agree

④ Disagree

⑤ Strongly disagree

⑥ Not sure

Q4. What do you anticipate being the top benefits of AI-H technology in the next five years? Please rank the top three in order of your expectations (1st, 2nd, 3rd).

① Discovery of the causes of diseases such as cancer and rare or incurable diseases

② Development of treatments for diseases such as cancer and rare or incurable diseases

③ Improvement in the accuracy of Interpreting medical imaging data (e.g., chest X-rays, MRIs)

④ Enhanced use of personal electronic medical record websites in emergencies (e.g., access to personal medical history not available to medical institutions)

⑤ Recommendations for antibiotics or painkillers based on specific situations

⑥ Early prediction of cancer occurrence

⑦ Diagnosis and notification of cancer occurrence

⑧ Providing regular healthcare for the elderly and disabled

⑨ Enhancing the international competitiveness of the domestic bio industry

⑩ Other (please specify):

Q5. What concerns do you have about healthcare AI technology in the next five years? Please rank the top three in order of your concerns (1st, 2nd, 3rd).

① Concerns about the disclosure of personal health-related information (e.g., childbirth, medication, illness)

② Concerns about monitoring genetic traits (e.g., DNA) in individuals and their relatives

③ Concerns about reduced access to human doctors for consultations

④ Concerns about potential harm caused from AI diagnostic errors

⑤ Concerns about the lack of understanding or explanation in AI decision-making due to unclear AI processes

⑥ Concerns about difficulties in identifying and understanding damages resulting from AI errors

⑦ Concerns about ambiguities regarding legal duties for AI-caused damages

⑧ Concerns about AI exacerbating medical disparities

Q6. Are you willing to utilize AI-H technology in the future?

① Strongly agree

② Agree

③ Disagree

④ Strongly disagree

⑤ Not sure

Q7. Are you willing to provide the following information for use in AI-H technology in the future?

Q7-1. Electronic medical records

① Strongly agree

② Agree

③ Disagree

④ Strongly disagree

⑤ Not sure

Q7-2. Genetic data

① Strongly agree

② Agree

③ Disagree

④ Strongly disagree

⑤ Not sure

Q7-3. Biometric data collected by smartphones or wearable devices

① Strongly agree

② Agree

③ Disagree

④ Strongly disagree

⑤ Not sure

Q7-4. Lifestyle data collected by smartphones or wearable devices

① Strongly agree

② Agree

③ Disagree

④ Strongly disagree

⑤ Not sure

II. Awareness of Ethics Principles of AI-H

The ethical principles of AI-H encompass the ethical considerations that must be addressed throughout all stages of AI technology in healthcare. This includes research and development, as well as the application, use, and distribution of the technology in real-world healthcare settings.

Q8. How familiar are you with the Ethics Principles of AI-H?

① I know it well.

② I know it to some extent

③ I've heard of it, but I don't know much about it

④ I've never heard of it

Q9. Do you agree that ethical principles are necessary when developing and utilizing healthcare artificial intelligence technology?

① Strongly agree

② Agree

③ Disagree

④ Strongly disagree

⑤ Not sure

Q10. Which groups do you think ethics education is necessary for? (Please select all that apply)

① Researchers

② Developers

③ AI-H Users

④ Students

⑤ The lay public

⑥ Data managers in hospitals

⑦ Data managers in governments (e.g., National Health Insurance Corporation)

⑧ Data managers in corporations (e.g., Marketing managers of AI-H companies)

⑨ Others (please specify):

Q11. Various ethical principles are proposed for consideration in the development and use of AI-H technology. How important do you think each of the following ethical principles is? Please rate each principle on a scale from 1 to 10, where 1 is 'Not very important' and 10 is 'Very important'.

Q11-1. Protection of Human Autonomy

- AI-H research should not infringe on individuals' rights to make their own decisions.
- Individuals should be able to participate in AI research and development involving their data.

Q11-2. Ensuring informed consent

- If the data subject provides data and specifies that it will be used only for a specific purpose (e.g., public interest research) and agrees to this condition, researchers and developers must use the data exclusively for that specified purpose.

Q11-3. Ensuring human oversight

- Decision-making authority must remain with humans, even in an automated healthcare environment.

Q11-4. Promotion of Purposeful Profit

- AI-H research should enhance human health, happiness, safety, and public interest.

Q11-5. Prevention of Harm

- AI-H research should uphold universal human rights. In addition, such research should make efforts to anticipate and prevent potential harm in advance.

Q11-6. Protection of Privacy

- The personal information of data subjects must be protected, and data collection, management, use, and disposal must follow established legal procedures.

Q11-7. Promotion of Safety and Security

- AI-H technology must prioritize safety. AI-H researchers and developers must be aware of security vulnerabilities such as hacking in advance and make every effort to prevent them.

Q11-8. Ensuring transparency

- The use of data in AI-H development should be based on social consensus, and research results utilizing large-scale datasets (e.g., public data) should be published periodically.

Q11-9. Ensuring explainability

- Researchers and developers should provide explanations of AI-generated results in understandable terms when requested by users.

Q11-10. Ensuring Reliability

- AI-H research must undergo rigorous verification and evaluation by independent organizations.

Q11-11. Upholding Accountability

- Researchers and developers participating in AI-H research must recognize their responsibilities regarding data utilization and AI development and act accordingly.

Q11-12. Upholding legal duties

- If harm occurs to individuals due to the use of AI-H, compensation and relief measures must be determined in advance. In addition, all parties must strive to minimize the damage.

Q11-13. Promoting inclusivity

- AI-H technologies should be applied regardless of individual characteristics such as age, gender, income, and ability.

Q11-14. Ensuring accessibility

- AI-H researchers, developers, service providers, and governments should make efforts to make AI technologies easily understandable and do their best to prevent the digital divide (differences in digital literacy between population groups) from widening further.

Q11-15. Elimination of discrimination

- Data should not benefit or discriminate against any specific individual or group.

Q11-16. Fostering responsiveness

- The company and government should establish institutional procedures to address and resolve complaints related to AI-H technology.

Q11-17. Fostering sustainability

- AI-H should be developed to reduce disparities in healthcare resources and minimize environmental burden.

Q12. Which ethical principle do you think is most important for each of the following AI-H technology use cases? Please read the description of each technology carefully and respond.

Q12-1. The following is a description of a technology called 'diagnosis assistance AI'. Please read the description and choose three ethical principles in order of which you think are most important.

**Diagnostic assistance AI** is software that helps doctors make diagnoses in medical settings, primarily used in imaging diagnoses such as X-rays, CT scans, and MRIs.

① Protection of human autonomy
② Ensuring informed consent
③ Ensuring human oversight
④ Promotion of purposeful profit
⑤ Prevention of harm
⑥ Protection of privacy
⑦ Promotion of safety and security
⑧ Ensuring transparency
⑨ Ensuring explainability
⑩ Ensuring reliability
⑪ Upholding accountability
⑫ Upholding legal duties
⑬ Promoting inclusivity
⑭ Ensuring accessibility
⑮ Elimination of discrimination
⑯ Fostering responsiveness
⑰ Fostering sustainability

Q12-2. The following is a description of the technology called 'doctor's decision assistance AI'. Please read the description and choose the three ethical principles in order of importance.

Doctor's decision assistance AI is a computer program that helps doctors in medical settings to decide on future treatment plans for patients' diseases. It is mainly used when doctors from various fields, including oncology, gather to make decisions and decide on treatment plans for patients' diseases.

① Protection of human autonomy

② Ensuring informed consent

③ Ensuring human oversight

④ Promotion of purposeful profit

⑤ Prevention of harm

⑥ Protection of privacy

⑦ Promotion of safety and security

⑧ Ensuring transparency

⑨ Ensuring explainability

⑩ Ensuring reliability

⑪ Upholding accountability

⑫ Upholding legal duties

⑬ Promoting inclusivity

⑭ Ensuring accessibility

⑮ Elimination of discrimination

⑯ Fostering responsiveness

⑰ Fostering sustainability

Q12-3. The following is a description of a technology called 'healthcare management AI'. Please read the description and choose three ethical principles in order of importance.

Healthcare management AI refers to providing real-time health management services to individuals by collecting biometric information (blood pressure, blood sugar, body composition, etc.) through wearable devices such as smartphones and smartwatches. Through this, personalized health management can be achieved, proper lifestyle habits can be developed, or chronic diseases can be managed.

① Protection of human autonomy

② Ensuring informed consent

③ Ensuring human oversight

④ Promotion of purposeful profit

⑤ Prevention of harm

⑥ Protection of privacy

⑦ Promotion of safety and security

⑧ Ensuring transparency

⑨ Ensuring explainability

⑩ Ensuring reliability

⑪ Upholding accountability

⑫ Upholding legal duties

⑬ Promoting inclusivity

⑭ Ensuring accessibility

⑮ Elimination of discrimination

⑯ Fostering responsiveness

⑰ Fostering sustainability

Q12-4. The following is a description of the technology called 'Treatment Assistance AI'. Please read the description and choose three ethical principles in order of importance.

**Treatment assistance AI** refers to assisting medical personnel in performing operations, treatments, and other activities for patients. Specifically, laparoscopic surgical robots and robotic surgical support AI are being utilized in the field. Additionally, technologies such as robot-assisted walking therapy using rehabilitation robots are being utilized.

① Protection of human autonomy

② Ensuring informed consent

③ Ensuring human oversight

④ Promotion of purposeful profit

⑤ Prevention of harm

⑥ Protection of privacy

⑦ Promotion of safety and security

⑧ Ensuring transparency

⑨ Ensuring explainability

⑩ Ensuring reliability

⑪ Upholding accountability

⑫ Upholding legal duties

⑬ Promoting inclusivity

⑭ Ensuring accessibility

⑮ Elimination of discrimination

⑯ Fostering responsiveness

⑰ Fostering sustainability

Q12-5. The following is a description of the technology called 'healthcare consultation AI'. Please read the description and choose three ethical principles in order of importance.

**<u>Healthcare consultation AI</u>** is a technology that comprehensively collects and utilizes personal health information, medical institution treatment information (medical records), and public institution information (national health insurance subscription information, vaccination status, surrounding environment records, infectious disease transmission information, etc.) to provide consultation on issues or questions related to an individual's health by AI, rather than a medical professional or healthcare professional.

① Protection of human autonomy

② Ensuring informed consent

③ Ensuring human oversight

④ Promotion of purposeful profit

⑤ Prevention of harm

⑥ Protection of privacy

⑦ Promotion of safety and security

⑧ Ensuring transparency

⑨ Ensuring explainability

⑩ Ensuring reliability

⑪ Upholding accountability

⑫ Upholding legal duties

⑬ Promoting inclusivity

⑭ Ensuring accessibility

⑮ Elimination of discrimination

⑯ Fostering responsiveness

⑰ Fostering sustainability

Q13. Do you think there are additional ethical principles that should be considered in addition to the ethical principles for AI-H mentioned above?

① Yes ☞ Go to Q13-1

② No ☞ End of question, go to SQ1

③ Don't know ☞ End of question, go to SQ1

Q13-1. (Only respondents who answered ① to Q13). What ethical principles do you think should be considered specifically? Please write in detail.

(Specifically)

III. Social and demographic characteristics

DQ1. What is your highest level of education?

① Less than middle school graduate

② High school graduate

③ College graduate

④ Graduate school or higher

DQ2. What is the average monthly income of your household (or family)?

① Less than 2 million KRW

② 2 million KRW or more, but less than 4 million KRW

③ 4 million KRW or more, but less than 6 million KRW

④ 6 million KRW or more

⑤ Not sure/Rejected

DQ3. Which of the following portable electronic devices do you own? (Multiple responses possible)

① Smartphone

② Smartwatch (e.g. Galaxy Gear, Apple Watch, etc.)

③ Tablet PC (e.g. Galaxy Tab, iPad, etc.)

④ None

DQ4. Have you ever used a wearable device (e.g. Fitbit, Apple Watch, Galaxy Watch)?

① Yes

② No

DQ5. Have you ever used a health-related apps (e.g. Apple Health, Samsung Health, etc.)?

① Yes

② No

DQ6. Have you ever used a Social Network Service (e.g. Facebook, Twitter, Instagram, KakaoTalk-linked service, etc.)?

① Currently using it

② Not currently using it, but have used it in the past

③ No

DQ7. How many times do you visit a medical institution per year?

① Once or less

② 2~5 times

③ 6~10 times

④ 11~15 times

⑤ 16~20 times

⑥ 21 times or more
